# Gene prediction in metagenomic fragments based on the SVM algorithm

**DOI:** 10.1186/1471-2105-14-S5-S12

**Published:** 2013-04-10

**Authors:** Yongchu Liu, Jiangtao Guo, Gangqing Hu, Huaiqiu Zhu

**Affiliations:** 1 State Key Laboratory for Turbulence and Complex Systems and Department of Biomedical Engineering, College of Engineering, Peking University, Beijing 100871, China; 2 Center for Theoretical Biology, Peking University, Beijing 100871, China; 3 Center for Protein Science, Peking University, Beijing 100871, China; 4 Laboratory of Molecular Immunology, National Heart, Lung and Blood Institute, National Institutes of Health, Bethesda, Maryland 20892, USA

## Abstract

**Background:**

Metagenomic sequencing is becoming a powerful technology for exploring micro-ogranisms from various environments, such as human body, without isolation and cultivation. Accurately identifying genes from metagenomic fragments is one of the most fundamental issues.

**Results:**

In this article, we present a novel gene prediction method named MetaGUN for metagenomic fragments based on a machine learning approach of SVM. It implements in a three-stage strategy to predict genes. Firstly, it classifies input fragments into phylogenetic groups by a *k*-mer based sequence binning method. Then, protein-coding sequences are identified for each group independently with SVM classifiers that integrate entropy density profiles (EDP) of codon usage, translation initiation site (TIS) scores and open reading frame (ORF) length as input patterns. Finally, the TISs are adjusted by employing a modified version of MetaTISA. To identify protein-coding sequences, MetaGun builds the universal module and the novel module. The former is based on a set of representative species, while the latter is designed to find potential functionary DNA sequences with conserved domains.

**Conclusions:**

Comparisons on artificial shotgun fragments with multiple current metagenomic gene finders show that MetaGUN predicts better results on both 3' and 5' ends of genes with fragments of various lengths. Especially, it makes the most reliable predictions among these methods. As an application, MetaGUN was used to predict genes for two samples of human gut microbiome. It identifies thousands of additional genes with significant evidences. Further analysis indicates that MetaGUN tends to predict more potential novel genes than other current metagenomic gene finders.

## Background

Thousands of prokaryotes have been cultivated and sequenced to explore the extent of biological diversity of the microbial world [1]. However, studies based on 16S ribosomal RNA approaches estimate that only a small fraction of the living microbes can be easily isolated and cultivated in laboratory conditions, thus single genome sequencing is not applicable for the majority of microbial species [2,3]. It means that the current knowledge of genomic data is highly biased and do not represent the true picture of the microbial species [4]. In addition, single genome sequencing ignores the interactions such as coevolution and competition between organisms living in the same habitats, which fail to reveal the real state of microbial organisms in nature.

These limitations can be circumvented by metagenomics, a methodology for studying microbial communties by directly sampling and sequencing shotgun DNA fragments from their natural environments without prior cultivation [5]. It is becoming a powerful method to reveal genomic sequences from organisms in natural environments, especially for communities resided in or on human bodies that are closely related to human health. With the evolutionary development of sequencing technologies, DNA sequences can be produced at much higher throughput with much lower prices than before. So far, hundreds of samples from various environments, such as, acid mine drainage [6], Sargasso sea [7], Minnesota soil [8] and human gut microbiome [9-11] have been sequenced by traditional Sanger sequencing and the next-generation sequencing (NGS) technologies like Roche454 and Illumina.

Accurate gene prediction is one of the fundamental steps in all metagenomic sequencing projects. However, it is more complicated in metagenomes than in isolated genomes. Firstly, most fragments are very short. Many sequences in metagenomic sequencing projects remain as unassembled singleton reads or short-length contigs. Therefore, lots of genes are incomplete with one or two ends exceed the fragments, which is not a problem in complete genomes. Also, a single fragment usually contains only one or two genes, non-supervised methods for single genomes which require an adequate number of genes for model training are inapplicable for this situation [12]. Secondly, the anonymous sequence problem, which means the source genomes of the fragments are always unknown or totally new [13,14], brings challenge on statistical model construction and feature selection.

Two types of approaches are commonly used for predicting genes from metagenomic DNA fragments. One is the evidence-based method that relies on homology searches. It includes comparisons against known protein databases by BLAST packages, CRITICA [15] and Orpheus [16]. Usually, it is able to infer functionalities and metabolic pathways of the predicted genes via significant targets with a high specificity if the threshold is stringent. However, only the genes with previously known homologs can be predicted by this means, while the novel genes, which are very important to metagenomic studies, will be overlooked. Therefore, *ab initio *algorithms that can present much higher sensitivity along with sufficient high specificity are indispensible.

Despite the anonymous and short fragmentary nature of sequences, several *ab initio *methods have been specially designed for metagenomic fragments in recent years [12-14,17-20], reporting that the performance on 3' end of genes is comparable with it on single genomes. Most of these previous methods based on modeling sequences in a Markov architecture of various orders. For example, MetaGeneMark incorporates a hidden Markov model to depict the dependencies between the frequencies of oligonucleotides with different length and the GC% of a nucleotide sequence by using direct polynomial and logistic approximations. It is found that the fifth-order Markov model obtained by logistic regression of hexamer frequencies performs the best [19]. Glimmer-MG was developed based on the Glimmer framework, which uses the interpolated Markov models with variable-order for capturing sequence compositions of protein-coding genes [14]. Orphelia is a recently proposed metagenomic gene finder based on the machine learning approach that by pass the Markov model [18]. It integrates mono-codon and di-codon usage, sequence patterns around TISs, ORF length and GC content into an artificial neural network to estimate the probability of an ORF to be protein-coding.

To overcome the anonymous sequence problem, MetaGene and MetaGeneMark train separate models for Archaea and Bacteria as studies have shown that the dependency patterns of oligonucleotides from GC content are different in the two domains of life [12,19]. An incoming fragment will be predicted by both models and the one with the higher score is chosen. In MetaProdigal, current complete genomes are firstly classified into 50 clusters according to the gene prediction similarity of Prodigal training files. Then, these clusters are used for learning another 50 training files for gene prediction in metagenomic fragments. A given fragment will be scored by the training files within a range of its GC content [13]. Glimmer-MG reported that the integration of sophisticated classification and clustering schemes based on interpolated Markov models to parameterized gene prediction models produces much better results than using GC-content [14]. In one of our previous works, MetaTISA introduced a *k*-mer method for binning sequences before TIS relocating. It also works well to achieve substantial improvement for TIS prediction [21]. In this article, we present a novel gene prediction method MetaGUN for metagenomic fragments based on a machine learning approach of support vector machine (SVM). Three sets of statistics are integrated to depict the coding potential for a candidate ORF, the EDP of codon usage, the TIS scores and the ORF length. The triplet nucleotides pattern is one of the most important statistic properties for discriminating protein-coding sequences from non-coding DNA. Different from most of the current metagenomic gene finders, MetaGUN describes the codon usage of ORFs by using an EDP model instead of the Markov model. The EDP model was used to measure the coding potential of ORFs based on the amino acids usage for single genomes in our previous works [22,23]. To be more sophisticated, the EDP model is extended to base on the codon usage for metagenomic fragments. Sequence patterns around TISs are also important signatures that can improve gene prediction performance [13,18,23]. In this work, we implement a TIS scoring strategy based on hundreds of precomputed TIS parameters trained by the TriTISA program to get the TIS scores for a given ORF [24]. The length of an ORF is the third integrated feature that has been reported to be another important measure for distinguishing genes from random ORFs in both isolated and metagenomic genomes [12]. Recently, special efforts have been made in predicting correct TISs by some current metagenomic gene finders with substantial achievements [13,14]. In MetaGUN, an upgraded version of MetaTISA is employed for adjusting the TISs for predicted genes. To identify protein-coding sequences, MetaGun builds two gene prediction modules, the universal module and the novel module. The former is based on 261 prokaryotic genomes representatively covering a wide range of phylogenetic clades, genomic GC content and varied living environments. The latter is designed to find potential functionary DNA sequences with conserved domains.

MetaGUN is freely available as open-source software from http://bioinfo.ctb.pku.edu.cn/MetaGUN/ under the GNU GPL Licenses.

## Materials and methods

### Data sets

Genomic data and annotations of 261 complete genomes (229 bacteria and 32 archaea) are obtained from NCBI RefSeq database for training the supervised SVM classifiers and the fragments classification model. 12 species (9 bacteria and 3 archaea) used in previous methods are also chosen for evaluating the prediction performance here [12,18]. Since the genomes of the 12 species are included in the training set, it is worth noting that we excluded them from the training data when assessing the performance on these genomes. The 6 genomes with experimentally characterized gene starts are used for evaluating TISs accuracy [21]. Two samples of human gut microbiome are used for investigating novel gene discovery ability of current methods [9]. Genomic sequences and corresponding annotations of them are obtained from IMG/M website.

### Architecture of MetaGUN algorithm

To predict genes, MetaGUN runs in three stages. Firstly, a *k*-mer based naïve Bayesian sequence binning method is employed to assign all incoming fragments into phylogenetic groups just like in our previous work MetaTISA [21]. In MetaGUN, it is worth noting that fragments are assigned into both the genus level and the domain level (Archaea and Bacteria). The former is used for supervised TIS scoring parameters selection and TIS prediction, and the latter is applied to determine the SVM classifiers for gene prediction. Secondly, all possible ORFs (complete and incomplete) are extracted from the fragments and scored by their feature vectors with SVM classifiers of supervised universal prediction module and sample specific novel prediction module for each domain independently. That is, a regressive probability is assigned to an ORF depending on its distance from the separating hyperplane in the feature space of the SVM classifier [25]. The ORF with a probability larger than the given threshold is regarded as protein-coding. Finally, a modified version of MetaTISA is used to relocate the TISs of all predicted genes to obtain high quality TIS annotations.

### Fragment classification

Since fragments in metagenomes can originate from diverse species, one of the most challenges is how to train statistical models that can properly capture features of sequences from different source genomes. Moreover, the short nature of metagenomic fragments further complicates this problem. Most published gene finders for metagenomes incorporate a sequence classification procedure implicitly or explicitly. For example, MetaGene and MetaGeneMark train separate models for two domains. Since they are based on the Markov model, input sequences are assigned to the domain whose model gives a higher score implicitly while predicting [12,19].

We employ a *k*-mer method based on a naïve Bayesian classifier for sequence binning before gene prediction [26]. The binning model is trained on complete sequences of the selected 261 genomes by calculating the frequencies of *k*-mer oligonucleotides for each of them. For a given fragment *s *with the length of *n *bases, the probability of finding it in one of the 261 genomes can be calculated according to the overlapping (*n-k*+1) oligonucleotides by using Bayesian classification. Then, the fragment *s *is regarded as originating from the genome with the highest poster probability (details see Additional file 1: Fragment classification strategy). It has been successfully implemented in our previous work MetaTISA [21]. To predict genes, we follow the strategy to train separate gene prediction models for Archaea and Bacteria that MetaGene and MetaGeneMark have applied. Therefore, the fragments will be also clustered into two different domains according to the phylogenetic relationships of the assigned genomes, and predicted by corresponding gene prediction models independently.

### Feature selection for SVM

The support vector machine approach has been widely used in solving prediction problems in bioinformatics that can be represented in the form of a binary classification, such as gene identification, protein-protein interaction prediction and horizontally transferred gene detection [27-29]. It can learn more accurate classifiers for patterns that cannot be easily separated in the input space by transforming the input patterns into a feature space using a suitable kernel function (details see Additional file 1: SVM algorithm in MetaGUN). Selecting relevant features for machine learning approaches is important for a number of reasons such as generalization performance, running efficiency and feature interpretation. The support vector machine method makes no exception. In this work, we utilize three sets of statistics to elucidate the coding potential, the EDP description of codon usage, the TISs scores and the ORF length.

#### EDP description of codon usage

The difference of sequence composition is the primary feature for discriminating protein-coding genes from non-coding sequences. This statistical property has been frequently used for gene prediction of prokaryotic genomes for a long history including both the isolated genomes and the metagenomes [12-14,18-20,23,30,31]. In our previous works of gene prediction in complete genomes, the EDP model was used to describe the global properties of ORFs for calculating the coding potential on the basis of the amino acid usage [22,23]. Its success validates the hypothesis that the protein-coding genes distribute separately from the non-coding ORFs in the EDP phase space, which may be caused by different selection pressures during the evolution [23]. To be more sophisticated, the EDP model was extended to be based on the 61-dimension codon usage and was found to be more accurate. So that the EDP $\left\{s_{i}\right\}$ of an ORF in this article is defined as:

(1) $$s_{i}=-\frac{1}{H}c_{i}logc_{i}$$

where $c_{i}$ is the abundance of the *i*th codon obtained by counting the number of it in the sequence divided by the total number of codons, *i *= 1, 2, ..., 61 represents the index of the 61 codons (excluding 3 stop codons), and $H=-\sum_{i=1}^{61}c_{i}logc_{i}$ is the Shannon entropy.

#### Translation initiation site scores

**Figure 1:**
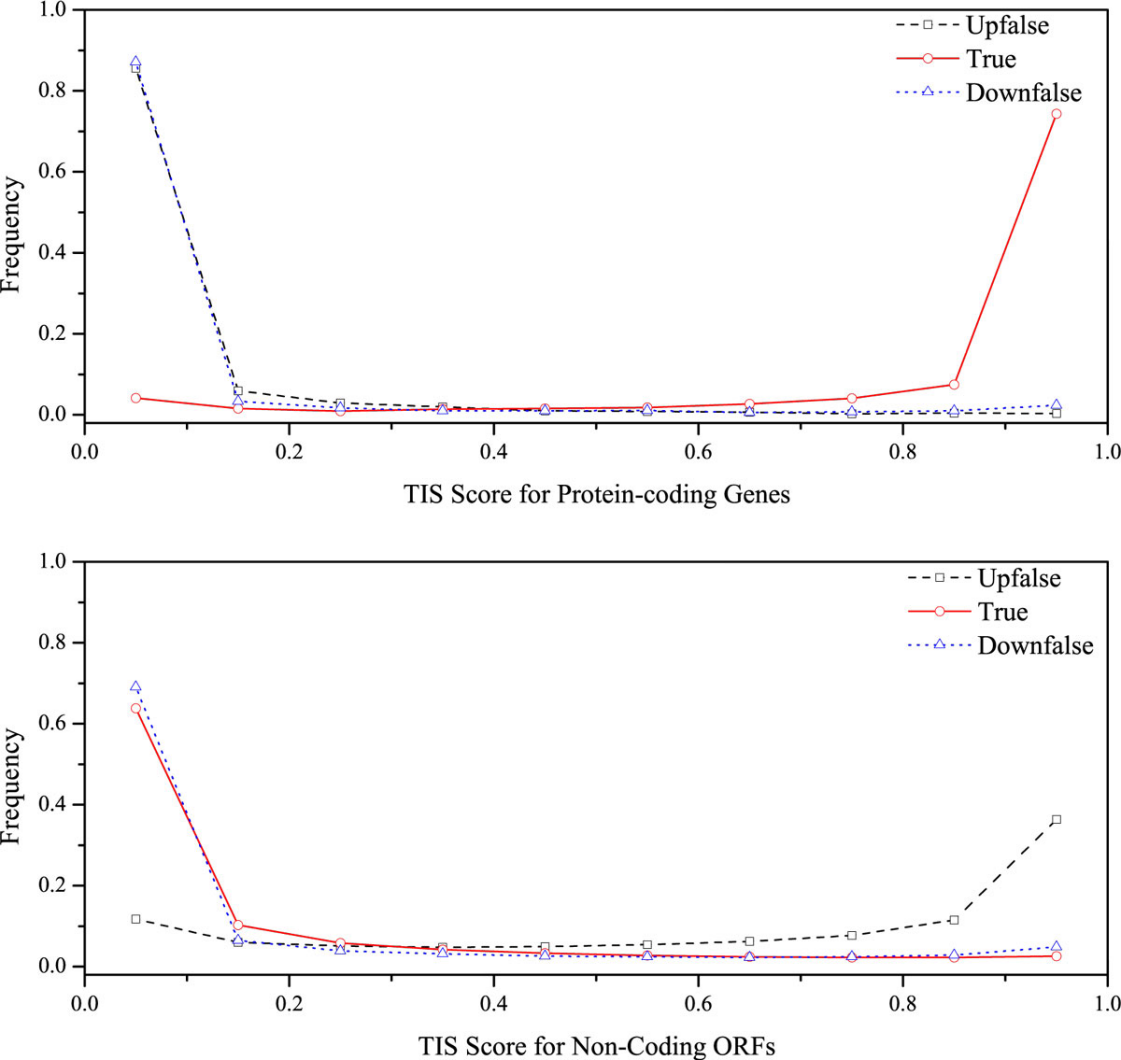
**The distributions of TIS scores of protein-coding genes (the upper one) and non-coding ORFs (the lower one)**. We simulated shotgun sequences by randomly sampling DNA fragments from *E. coli *K12 genomic sequence with fixed-length of 870 bp. Upfalse, True and Downfalse are stand for the probabilities of a TIS to be the candidate TIS from non-coding region, to be the true TIS and to be the candidate TIS from coding region, respectively.

The common motifs and surrounding sequences around the TISs are also important signatures of protein-coding genes [13,18,23]. To integrate this feature into MetaGUN, we implement the MetaTISA algorithm in a supervised manner to get the TIS scores. For each candidate TIS in an ORF, the probabilities to be the true TIS $\left(P_{t}\right)$, to be the start codon from non-coding region $\left(P_{nc}\right)$ and to be the start codon from coding region $\left(P_{co}\right)$ are estimated by MetaTISA according to the precomputed TIS parameters of the 261 training genomes. The choice of the TIS parameters are determined by the fragment classification results of the genus level. The one with the highest $P_{t}$ will be regraded as the predicted TIS in this stage, and the three probabilities of this TIS are treated as the TIS scores of the ORF. Figure 1 shows the distinguished distributions of the three TIS scores in protein-coding genes and non-coding sequences of artificial fragments sampled from *Escherich coli *K12. However, note that many ORFs in metagenomic fragments are incomplete with no leftmost candidate starts or even no candidate starts for the short lengths. To avoid complicating the problem by estimating whether the true TISs run off the edges of the fragments or not, we simply construct separate models for these two types of ORFs. That is, the TIS scores are ignored for the ORFs with incomplete 5' ends. Actually, the true TISs of genes with missed 5' ends are not included in the fragments in most cases because TIS prefers to be the leftmost of a gene [23,24].

#### The length of ORFs

The ORF length is another useful feature that has been frequently used for the discrimination of protein-coding and non-coding ORFs [12,14,18,31]. It is reported that the average length of genes in complete genomes is about 950 bp, which is much longer than random ORFs [12]. In some current methods, a log-odds score or log-likelihood ratio is assigned to a given ORF according to the distributions of protein-coding genes and non-coding ORFs that are trained on complete genomes [12,14]. However, the difficulty in integrating the ORF length feature is that a lager number of ORFs are incomplete for the short nature of metagenomic fragments [12,14]. This phenomenon indicates that the complete and the incomplete ORFs should be treated separately. Since MetaGUN is built on a machine learning approach of the SVM, it is very convenient to accomplish the complete and incomplete issues in ORF length for they can be treated as two separate features. Hence, two values are assigned as ORF lengths, one for complete and the other for incomplete. For a specific ORF, the value of the corresponding type is set as the actual ORF length, while the other value is set to zero.

The composition patters of sequences from archaeal and bacterial genomes have been reported to be different, and tests have shown that the prediction scores will be degraded if models from the wrong domain are employed for scoring [12,19]. Therefore, separate SVM classifiers for Achaea and Bacteria are trained on corresponding training genomes to server as gene prediction models in MetaGUN.

### Gene prediction model training

To identify protein-coding genes, MetaGUN comprises two gene prediction modules namely the universal module and the novel module. SVM classifiers of the universal gene prediction module are trained based on complete genomes with the purpose of capturing the universal features of current known genes. In this work, to build the universal prediction module, 261 species are selected from NCBI RefSeq database release 45 (the latest release version at the time we started to design MetaGUN algorithm) according to the 'one species per genus' rule [12]. The selected 261 species cover a wide range of phylogenetic clades, GC content and are isolated from varied environmental conditions, which can serve as good representatives for sequenced microbes. The amount of sequenced complete microbial genomes is growing dramatically with the revolutionary development of sequencing technology, however, we have found that our method based on these training genomes performs good results (see Results and discussions), which indicates that the selection of training genomes do capture the universal features of current known genomes. Moreover, many metagenomic sequencing projects aim to study the unculturable microorganisms, whose complete genomic sequences are currently unavailable. In these studies, the discovery of new genes with novel functionality is one of the principle objectives [32]. Methods have been developed for the detection of the novel genes based on searching for conserved domains against known databases [32,33]. The domain-based searches have been reported to be more sensitive to target genes than sequence similarity based methods like BLASTP because conserved domains other than the whole sequences are compared [27,34]. For instance, Bork *et al. *applied the conserved domain analysis to RcaE proteins, and predicted 16 novel domain architectures that may have potential novel functionalities in habitats with little or no light [32]. In our work, in an effort to address the novel gene prediction issue, a sample specific novel prediction module based on domain searches is incorporated.

#### Universal prediction module

To train SVM classifiers of the universal gene prediction module, artificial shotgun fragments are randomly sampled from the complete genomic sequences for each of the 261 training genomes by MetaSim to form 3x coverages [35]. We generate fragments with lengths ranging from 60 bp to 1500 bp in order to simulate DNA sequences from different sequencing technologies. Then, all complete and incomplete ORFs are extracted from these fragments and represented as input feature vectors for training SVM classifiers. Those can originate from the annotated genes are used as training instances of protein-coding class, whereas others are treated as items of non-coding class. ORFs less than 60 bp are ignored, for they are too short to provide useful information. The training data of Bacteria and Achaea are constructed by mixing together the feature vectors of ORFs from the same domains, and SVM classifiers are then trained independently. Different types of discriminatory functions can be learned by the SVM algorithm with the combination of a number of kernel functions, such as linear kernel, polynomial kernel and Gaussian kernel. Meanwhile, the performance usually gets better if more training items are included, however, the training time grows exponentially along with the size of training data. Since the amount of training items in each domain is large, especially for Bacteria because hundreds of species are involved, we need to learn sufficient good classifiers with proper training size, as well as finding the most suitable kernel function for metagenomic gene prediction. Hence, experiments are carried out to evaluate the prediction accuracies on simulated fragments of the 12 testing genomes, with SVM classifiers trained on different kernel functions and various training data size. The results (see Additional file 1: Supplementary Table 1) show that the non-linear kernels (polynomial and Gaussian) behavior much better than the linear kernel, and between non-linear kernels, the performance on Gaussian kernels are slightly better. Meanwhile, we find that 1.6 M is a proper training size of both sufficient and efficient since the observed accuracy improvements brought by larger training size are marginal. Therefore, in this stage, a subsets of training data is randomly sampled into 1.6 M for each domain to train SVM classifier with Gaussian kernel function separately.

**Table 1:** Gene prediction performance on simulated shotgun sequences.

Methods		1200 bp			870 bp			535 bp			120 bp	
	Sn(%)	Sp(%)	Hm(%)	Sn(%)	Sp(%)	Hm(%)	Sn(%)	Sp(%)	Hm(%)	Sn(%)	Sp(%)	Hm(%)
MG	97.7	**94.8**	**96.3**	97.4	**95.2**	**96.3**	96.9	**95.4**	**96.1**	93.2	**89.6**	91.4
MGC	98.0	**95.2**	**96.6**	97.7	**95.5**	**96.6**	97.2	**95.7**	**96.4**	93.3	**90.0**	**91.6**
MP	97.5	93.6	95.5	97.2	93.5	95.3	96.8	92.9	94.8	92.0	85.5	88.7
GLM	**98.1**	93.3	95.6	**97.9**	93.3	95.6	**97.7**	93.1	95.3	**94.7**	88.7	**91.6**
MGM	97.5	92.7	95.1	97.1	92.9	94.9	96.7	92.8	94.7	90.1	89.1	89.6
MGA	97.4	91.7	94.4	97.2	91.4	94.2	96.8	90.5	93.5	91.3	83.7	87.4
FGS	95.7	87.3	91.3	95.5	88.0	91.6	95.2	88.4	91.6	90.4	82.1	86.1
Net	94.6	94.7	94.6	94.1	94.7	94.4	93.3	94.6	93.9	82.0	76.4	79.1

The gene prediction methods are denoted by abbreviations. MG: MetaGUN, MGC: complete version of MetaGUN that trained on all 261 training genomes, MP: MetaProdigal, GLM: Glimmer-MG, MGM: MetaGeneMark, FGS: FragGeneScan, MGA: MetaGeneAnnotator, Net: Orphelia.

#### Novel prediction module

In the purpose of predicting genes that might be difficultly recognized by the universal gene prediction module, the sample specific novel module is then incorporated into MetaGUN based on the domain search approaches. Firstly, the extracted ORFs are translated into amino acid sequences and searched for conserved domains against the Conserved Domain Database (CDD) database. Those carrying detected domain motifs with significant *e*-values (< 10^-40^) are treated as training data of genes. To obtain the training instances of non-coding ORFs, we follow GISMO to implement the 'shadow' rule [33]. That is, an ORF overlapping more than 90 bp with a targeted gene in another reading frame is regarded as a non-coding ORF. Then, the training data is clustered into two phylogenetic groups of Archaea and Bacteria according to the fragments classification results, and is employed as input feature vectors for training SVM classifiers for each domain independently. If the size of training items is larger than 1.6 M, a subset of 1.6 M will be randomly sampled for training SVM classifier according to the experience in the universal prediction module; otherwise, the whole training set will be used.

LibSVM package is employed in our work to train the SVM classifiers with Gaussian kernel function for both the universal prediction module and the novel prediction module [25]. In each training procedure, a grid search of feature space is firstly implemented to find the most suitable Gaussian kernel parameter γ and SVM parameter *C *(details see Additional file 1: SVM algorithm in MetaGUN). Then all items in the training set of both the protein-coding and non-coding classes are implicitly mapped from the input space to the feature space that is determined by the Gaussian kernel under the learned best γ and *C*. Finally, a hyperplane (the SVM classifier) is learned by the SVM training program that optimally separates all training protein-coding and non-coding items.

### Translation initiation site prediction

Accurate gene starts prediction is also a very important issue in metagenomic sequencing projects which is indispensable for experimental characterization of novel genes, however, has not been studied much in the literature [13,21]. TIS prediction for complete genomes has a long history and a number of tools have been developed [24,36-41]. The difficulty of TIS prediction in prokaryotic genomes is the divergency of the regulatory signals which indicate divergent translation initiation mechanisms. Studies have revealed that in the upstream of the TISs there are SD motifs for leadered genes and Non-SD signals for leaderless genes [41-43]. However, the short and anonymous nature of metagenomic fragments present more challenges.

In one of our previous works, MetaTISA has been built to accomplish this problem and has greatly improved the TIS annotations for MetaGeneAnnotator [21]. Recently, two works have paid special attentions to the TIS prediction and have achieved substantial progresses [13,14]. For example, MetaProdigal follows the same strategy as Prodigal, its version for isolated genomes, to use a TIS scoring system that integrates default scoring bins based on prior RBS motifs and rigorous searches for alternative motifs if no SD motifs appears [13]. It also reported that the published MetaTISA tends to predict starts to downstream start codons for the genes whose true TISs are close to or run off the edge of the fragments [13].

**Figure 2:**
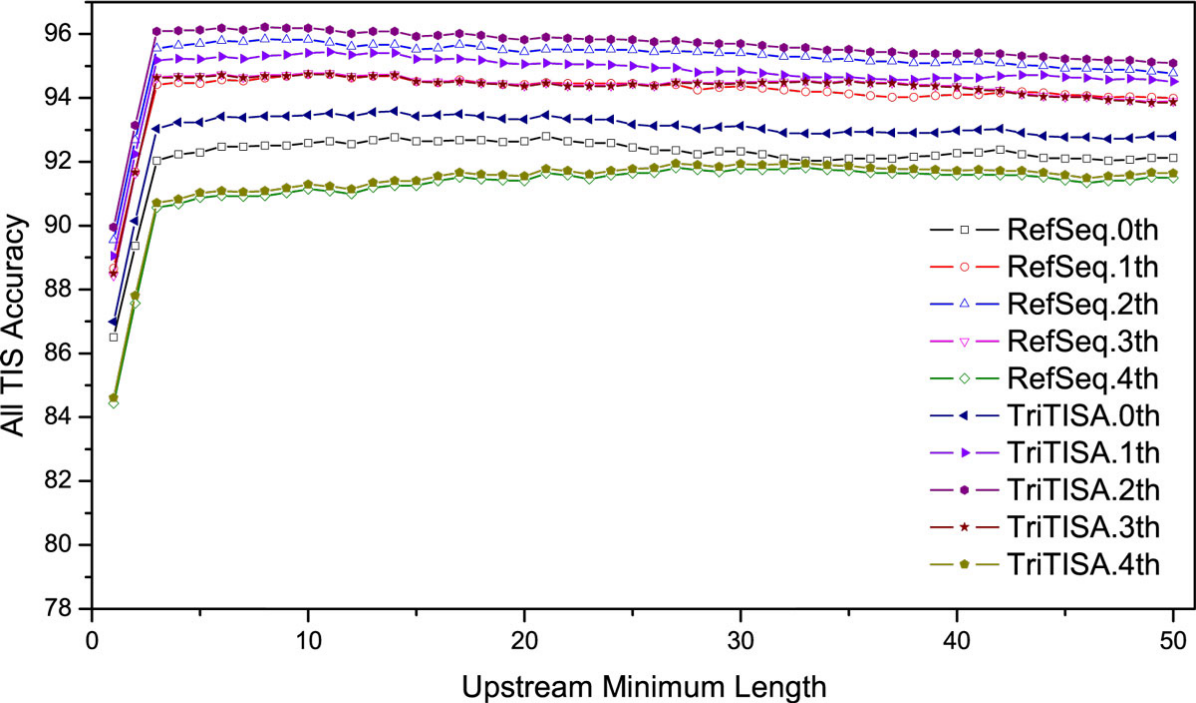
**TIS prediction experiments by modified MetaTISA on simulated shotgun DNA fragments**. The artificial shotgun sequences are sampled from *E. coli *K12 with fixed-length of 870 bp. The upstream minimum length means the minimum requisite amount of upstream bases used for scoring if it is less than 50 bp, and the TIS accuracy is the overall accuracy of both the internal and the external TISs. The supervised TIS parameters used for the experiments including those trained on the RefSeq annotations and the TriTISA annotations, with Markov models ranging from 0-order to 4th-order.

According to exhaustive analysis, we modify MetaTISA by amending two settings and the supervised TIS parameters when dealing with incomplete genes. In previous MetaTISA, the distribution of *P_co _*is used for estimating whether the 5' most candidate TIS is from coding regions or not for genes incomplete in their 5' ends [21]. However, it is too stringent to set the confidence level at 99%. Many candidate TISs actually locate in coding region are regarded as upstream candidates, and then the algorithm runs to find the false TISs downstream in the coding area. Tests on simulated sequences from *E. coli *K12 show that the threshold should be loosen to the confidence level at 95% to achieve the best results. Another practical problem for some genes is the insufficiency of upstream bases for TIS scoring. The published MetaTISA requires 50 bp upstream sequences of a candidate TIS to calculate the three poster probabilities. As a result, TIS candidates not satisfying this requirement will be overlooked. Experiments are performed to obtain the optimal value of the minimal requisition of upstream bases (Figure 2). Moreover, various orders of Markov models and the supervised TIS parameters that trained on different annotations (RefSeq and TriTISA) are investigated. Based on the performance shown in Figure 2, we determine to set the minimal requisite length of upstream sequence as 10 bp, the maximum order of Markov model to be 2 and all precomputed TIS parameters are trained on TriTISA annotated genes.

## Results and discussion

Due to the lacking of experimentally characterized genes and translation initiation sites in metagenomic sequencing projects, the performance of current methods are all evaluated on simulated fragments [12-14,18-21]. However, two significant drawbacks of this methodology should be noted. Firstly, most annotated genes in NCBI RefSeq and GenBank database have not been verified by experiments. Annotation errors have been reported in some species, especially for the genomes with high GC-content [44,45]. So, in recent studies of metagenomic gene finders, annotated hypothetical genes are removed from the benchmarks for reliable assessment [13,14,19]. Secondly, the reliability of TIS annotations in public databases is also suspicious. Large scale computational evaluation has been reported that RefSeq's TIS annotations biased to over-annotate the leftmost start codons and under-annotate the ATG start codons [46]. Here, in the performance comparison of gene prediction, we follow MetaGene and Orphelia to choose the 12 genomes which have a good coverage of Archaea and Bacteria, as well as varied levels of GC content. Considering the mentioned problems in RefSeq annotations, we follow the same strategy as MetaGeneMark to discard the fragments containing any annotated hypothetical genes [19]. Moreover, the TIS prediction accuracy are not evaluated on these genomes for the unreliability of TIS annotations. Instead, we use the 6 genomes where experimentally characterized gene starts are available for TIS prediction assessment [21].

### Gene prediction performance on artificial shotgun sequences

We compare the prediction performance of MetaGUN on 3' end of genes with 6 current metagenomic gene finders in this section. Artificial shotgun fragments with 3x coverage are simulated for each of the 12 testing genomes. To demonstrate sequences produced by different sequencing technologies, three kinds of simulation are created with different sequence lengths (870 bp, 535 bp and 120 bp) according to the settings in Glimmer-MG [14]. In addition, fragments with length of 1200 bp are also simulated in order to investigate the performance on assembled contigs of larger size. Predictions with exactly matched 3' ends or matched reading frame if 3' ends are missed are regarded as correctly predicted genes, that is, the true positives. The sensitivity (Sn) and the specificity (Sp) are defined as the true positives in all annotated genes and in all predicted genes, respectively. We also use the harmonic mean value as a composite measure of sensitivity and specificity, which is defined as 2 SnSp/(Sn+Sp). Note that unlike the comparisons in Glimmer-MG, simulated fragments overlapping annotated hypothetical genes are excluded from the testing sets in this work, hence the benchmarks are complete and the measures of sensitivity and specificity are both meaningful.

The predictions of other methods are obtained by local running. The 'complete' model parameter trained for error-free sequences is set to run FragGeneScan [20], and both the 'Net700' and 'Net300' model are used for running Orphelia and the better result is chosen for comparison [18]. Others are implemented by default settings. For comprehensive investigation, we run two versions of MetaGUN, one is trained on all 261 training genomes which denotes as 'MGC' in Table 1; the other is trained on genomes excluding 12 testing genomes which denotes as 'MG'. The comparisons with other methods is based on the 'MG' version. In addition, since most metagenomic gene finders overlook genes less than 60 bp, we only evaluated genes with length more than that.

The accuracies are shown in Table 1. For fragments of longer length, that is 1200 bp, 870 bp and 535 bp, MetaGUN outperforms other gene finders in harmonica mean with values over 96%. While for shorter fragments of 120 bp, performance falls severely for all methods, especially Orphelia. This illustrates one of the challenges for predicting genes on short sequences is the uninformative incomplete ORFs. At this length, MetaGUN and Glimmer-MG achieves comparable performance with more than 91% in harmonic mean, which is much better than other methods. It is worth noting that MetaGUN always makes the best specificities among all simulations with different fragment lengths, which means its prediction is the most reliable. The Orphelia method, the other one based on the machine learning approach, also exhibits good results in specificity in longer fragments. However, its sensitivities are usually lower than others. The comparison on the results of 3' ends indicates that MetaGUN makes better predictions among existed algorithms for longer fragments that are produced under Sanger and Roche454 sequencing platforms, as well as longer contigs after assembly. Despite the performance is not superior to Glimmer-MG on the shorter fragments corresponds to Illumina sequencing platform, it is still much better than others. Moreover, with the aid of deep sequencing and effective assembly, the length of contigs will get longer. In a recent study on human gut microbiome with deep sequencing, Qin *et al. *reported that as much as 42.7% of the Illumina GA reads have been assembled to contigs longer than 500 bp, with an N50 length of 2.2 kb [11]. Meanwhile, the sequencing technologies are developing to produce longer reads in which MetaGUN can perform better than others.

A practical problem of metagenomic fragments is the sequencing errors. The error rates of raw data are reported to range from 0.001% to 1% for Sanger sequencing, and from 0.5% to 2.8% for pyrosequencing [47]. Prior work has shown that sequencing errors present severe impact on gene prediction, especially the frame shifts [47]. Two of previously mentioned metagenomic gene finders, FragGeneScan and Glimmer-MG, have specially designed models to address this issue and have achieved better accuracies than other methods when running on error-prone fragments [14,20]. However, in this work, we concentrate on predicting genes on error-free fragments for following reasons. Firstly, most low-quality nucleotides locate around the ends of the reads, and can be cut out by quality trimming and vector screening, or can be corrected by sequence assembly [47]. Secondly, separate software has been developed for identifying frame shifts for metagenomic fragments. It can be implemented prior to gene prediction to reduce the influences of sequencing errors [48]. Moreover, it is promising that frame shift can be greatly decrease with the aid of deeper sequencing, effective assembly and future improvements of sequencing technologies.

### TIS prediction performance on experimental data

Since many environmental sequencing projects are aiming at studying gene functions by experimentally characterization, accurate prediction of TISs is very important for correct TISs is indispensable for expressing genes [18,21]. To investigate the TIS prediction performance, we implement almost the same strategy applied in MetaTISA with two adjustments. Firstly, we follow Hyatt *et al. *[13] to assess the TIS accuracy on both the internal TISs and the external TISs. An internal TIS is a TIS locates inside a fragment, and an external TIS is that exceeds the edge of a fragment. Secondly, the simulated fragment lengths are 870 bp and 535 bp. Shorter fragment is not considered in TIS assessment as it is too short that the true TIS exceeds the fragment in most cases.

The performance of TIS prediction is shown in Table 2, in which the accuracy is the ratio of correctly predicted TISs from successfully identified genes. Based on the results, MetaGUN achieves to correctly predict 96.1% of the TISs for both simulations, which is the best performance among current metagenomic gene finders. MetaProdigal and Glimmer-MG also predict TISs in a high accuracy at over 95%, due to the integration of TIS scoring module. In detail, MetaProdigal always shows the best results for external TISs; while MetaGUN has the highest accuracy for internal TISs which is much higher than others, and shows an average performance for external TISs. Since experimental characterization and sequence analysis around TIS for studying translation initiation mechanisms rely more on accurate position of internal TISs than invisible external TISs, the superiority of internal TISs by MetaGUN might have more biological significance.

**Table 2:** TIS prediction performance on experimentally characterized gene starts.

Methods	870 bp			535 bp		
	Total	Internal	External	Total	Internal	External
MG	**96.1%**	**93.5%**	98.5%	**96.1%**	**91.2%**	98.8%
MP	95.1%	90.1%	**99.8%**	95.6%	88.1%	**99.7%**
GLM	95.0%	91.2%	98.7%	95.4%	89.2%	98.8%
MGM	92.1%	84.3%	99.4%	93.4%	82.5%	99.4%
MGA	90.9%	82.3%	98.9%	92.4%	81.1%	98.6%
FGS	86.2%	72.8%	98.8%	89.4%	72.2%	98.9%
Net	84.3%	78.6%	89.8%	88.0%	72.4%	96.4%

The abbreviations of gene prediction methods are the same as in Table 1. We follow Hyatt *et al. *[13] to assess the TIS accuracy on both the internal TISs and the external TISs. An internal TIS is a TIS locates inside a fragment, and an external TIS is that exceeds the edge of a fragment. The total means the overall accuracy of both the internal and the external TISs.

### Application to human gut microbiome

In order to investigate the application on real environmental sequencing projects, two samples of human gut microbiome from two healthy humans are selected for analysis [8]. Each sample consists of around ten thousand contigs with an average length of about 950 bp. Gene annotations are obtained from the IMG/M website. The annotated genes are identified by both the automatic *ab inito *gene finding softwares such as fgenesb, Glimmer and GeneMark, and similarity comparison approaches like BLASTx running against known protein databases [30,36]. MetaGUN and 6 other gene finders are then applied to predict genes for both samples. Table 3 shows the analysis results. In both samples, most of the annotated genes are successfully predicted, with comparable coverages among different methods. Meanwhile, thousands of additional genes are predicted in each sample when compared to the annotations. To examine the reliability of the additional genes, similarity search by BLASTP are then carried out against NCBI non-redundant database. Genes with significant hits (*e-value <*10^-5^) are regarded as 'annotated missed genes'. Results show that MetaGUN and Orphlia predict less additional genes than other methods. However, on the aspect of the percentages of the annotated missed genes among all additional predicted genes, the results of MetaGUN are higher than others in both samples. It indicates that MetaGUN tends to produce more reliable predictions which are consistent with the assessments on simulated fragments. One of the principle objectives for metagenomic sequencing projects is the discovery of novel genes. However, due to the lacking of experimentally verified genes in real samples, it is a difficult task to obtain an comprehensive evaluation like assessments of the gene and the TIS predictions in previous sections. In this section, we are trying to provide a clue on novel gene discovery ability with the aid of domain-based searches. The domains are functional units within proteins, which are usually conserved as building blocks during molecular evolution. Sometimes, the arrangement of domains varies to form proteins of different functions [49]. Therefore, domain-based searches are more sensitive for catching novel genes than protein sequences based searches [27,34]. We define 'potential novel genes' as follows. Firstly, all possible ORF are extracted and translated into amino acid sequences for domain searching against CDD, those with targeted domain motifs with an *e *value less than 10^-5 ^are denoted as potential functional genes. The IMG/M annotated genes and the genes with targets in the NR database are treated as known genes. Then, a potential functional gene which is not a known gene is regarded as a potential novel gene. From Table 3, we can see that MetaGUN predicts the largest amount of potential novel genes in both samples benefit from the integration of novel prediction module. Further analysis are then carried out to infer probable functionality for potential novel genes predicted by our method according to the targeted domains. We find that most targeted domains originate from proteins in bacterial genomes. Such as, *infB*, corresponds to the translation initiation factor IF-2, which is different from the similar proteins in the Archaea and Eukaryotes and acts in delivering the initiator tRNA to the ribosome; PRK12678, corresponds to the transcriptional terminator factor Rho; as well as several domains from DNA polymerase like PRK05182, PRK12323. It seemed that these potential genes should be identified by most gene finders and the sequence based similarity searches since they are essential for the survival of bacteria. However, they are categorized as potential novel genes for two possible reasons. In one situation, the targeted domain belongs to a actual novel protein which also consists of multiple unknown domains with novel functionality. In the other situation, the targeted domain belongs to a known protein which is truncated and too short for the identification by other methods.

**Table 3:** Application to 2 human gut microbiome samples.

Samples	Size(M)	Contigs	Annotated	Methods	Predicted	Additional	Potential novel
				MG	21524 (94.8%)	2101 (58.1%)	**32**
				MP	22056 (96.3%)	2332 (54.1%)	5
				GLM	22116 (96.4%)	2361 (54.5%)	5
Sub. 7	15.8	10411	20487	MGM	22200 (96.8%)	2365 (56.7%)	5
				MGA	22102 (96.3%)	2377 (57.2%)	3
				FGS	23215 (95.6%)	3634 (34.9%)	4
				Net	21421 (94.5%)	2067 (48.7%)	3

				MG	26881 (95.0%)	2241 (64.5%)	**12**
				MP	27737 (97.0%)	2589 (61.6%)	5
				GLM	28127 (97.1%)	2931 (58.2%)	5
Sub. 8	20.5	12020	25943	MGM	27931 (97.1%)	2728 (63.7%)	4
				MGA	27627 (96.2%)	2666 (63.1%)	4
				FGS	29462 (96.5%)	4433 (36.0%)	4
				Net	26780 (95.0%)	2126 (58.0%)	4

In this experiment, Orphelia runs used 'Net700' parameter and FragGeneScan runs used 'complete' mode for sequences in these samples are highly assembled. Others run under default settings. Percentages in the column 'Predicted genes' are ratios of successfully predicted genes to annotated genes; and percentages in the column 'Additional genes' are the ration of annotated missed genes to additional genes.

It is widely accepted that microorganisms in human gut microbiome can contribute certain vitamins to the host [11]. We have found an interesting case that can provide a clue. A domain named *cobN*, which usually exists in *cobN *genes that involved in cobalt transport or B12 biosynthesis in a number of species like *actinobacteria*, *cyanobacteria*, *betaproteobacteria *and *pseudomonads*. Moreover, domains involved in short-chain dehydrogenase are also detected in some genes, which is reported to be used by gut bacteria for fermentation to generate energy and converting sugars [11]. Similar to the phylogenetic distribution of genes analysis on IMG/M website, domains originated from Eukaryotes and Viruses are also detected, like ATG13 (from Autophagy-related protein 13), *danK *(from heat shock protein) and PAT1 (from Topoisomerase II-associated protein).

## Conclusion

In this article, we present a novel method for identifying genes in metagenomic fragments. It comprises three steps for gene prediction by firstly classifying input sequences into different phylogenetic groups, then identifying genes for each group independently with both universal prediction module and novel prediction module and finally relocating TISs employing a modified version of MetaTISA. We compared the prediction results with 6 current metagenomic gene finders. For the performance on 3' end of genes, MetaGUN are better than other methods on longer fragments and are comparable with Glimmer-MG which are much better than others on shorter fragments. A notable advantage is that MetaGUN always makes the best reliable predictions. For the assessments of 5' end of genes, MetaGUN outperforms others on the overall TISs and especially predicts much more correct internal TISs. The application to 2 samples from human gut microbiome also shows that MetaGUN predict more reliable results. Furthermore, we have attempted to investigate the novel gene discovery ability on these 2 real samples. With the effective integration of the novel prediction module, MetaGUN can find more potential novel genes than others. Detailed analysis of the discovered potential novel genes shows that there exists a number of biological meaningful cases. Overall, MetaGUN makes substantial advances for gene prediction in metagenomic fragments with three notable contributions: the improvements for both the protein-coding sequences and the translation initiation sites, and the greater ability for novel gene discovery. We believe that MetaGUN will serve as a useful tool for both bioinformatics and experimental researches.

## Authors' contributions

HQZ and YCL, GQH conceived the study, YCL and JTG designed the algorithm and performed the simulations and data analysis, YCL drafted the manuscript, HQZ supervised the progress of the work. All authors read and approved the final manuscript.

## Competing interests

The authors declare that they have no competing interests.

## Supplementary Material

Additional file 1 **MetaGUN additional file**. This addition file consists of 3 parts. The first is the fragment classification strategy, which describes the detailed strategy of the Bayesian methodology based on a *k*-mer method. The second is the SVM algorithm in MetaGUN, which describes the SVM algorithm, its integration into metagenomic gene prediction and the training procedure of SVM classifier in our work. The third is supplementary table 1 which illustrates the performance of universal module with SVM classifiers trained on various training size and difference types of kernel functions. Click here for file

## Declarations

Publication of this article was supported by the National Key Technology Research and Design Program of China (2012BAI06B02), National Natural Science Foundation of China (30970667, 11021463, 61131003 and 91231119), National Basic Research Program of China (2011CB707500), and Excellent Doctoral Dissertation Supervisor Funding of Beijing (YB20101000102).

This article has been published as part of *BMC Bioinformatics *Volume 14 Supplement 5, 2013: Proceedings of the Third Annual RECOMB Satellite Workshop on Massively Parallel Sequencing (RECOMB-seq 2013). The full contents of the supplement are available online at http://www.biomedcentral.com/bmcbioinformatics/supplements/14/S5.
