# Supplementary material for: Gene prediction in metagenomic fragments based on the SVM algorithm
Source: BMC Bioinformatics. 2013 Apr 10;14(Suppl 5):S12. doi: 10.1186/1471-2105-14-S5-S12 (PMC3622649; doi:10.1186/1471-2105-14-S5-S12)
Supplement: Additional file 1 — MetaGUN additional file. This addition file consists of 3 parts. The first is the fragment classification strategy, which describes the detailed strategy of the Bayesian methodology based on a k-mer method. The second is the SVM algorithm in MetaGUN, which describes the SVM algorithm, its integration into metagenomic gene prediction and the training procedure of SVM classifier in our work. The third is supplementary table 1 which illustrates the performance of universal module with SVM classifiers trained on various training size and difference types of kernel functions. [file 1471-2105-14-S5-S12-S1.PDF]

Gene Prediction in Metagenomic Fragments Based on the SVM Algorithm – Additional File

Yongchu Liu[1,2], Jiangtao Guo[1,2], Gangqing Hu[1,2,4], and Huaiqiu Zhu[1,2,3,⋆]

[1]State Key Laboratory for Turbulence and Complex Systems and Department of Biomedical Engineering, College of Engineering, Peking University, Beijing 100871, China [2]Center for Theoretical Biology, Peking University, Beijing 100871, China [3]Center for Protein Science, Peking University, Beijing 100871, China [4]Laboratory of Molecular Immunology, National Heart, Lung and Blood Institute, National Institutes of Health, Bethesda, Maryland 20892, USA

Fragment classification strategy

The fragment classification model is learned from complete genomic sequences of the 261 training genomes. For a given k-mer motif, the occurrences of all overlapping motifs from each genome are counted by scanning the whole sequence. The motif frequency profiles table is employed as the fragment classification model. In our algorithm, the classification model with k=8 is used. For a given DNA sequence S with length n which consists of (n-k+1) overlapping k-mer motifs, the probability of finding S in genome G_i can be calculated as the product of (n-k+1) probabilities of finding the motifs in genome G_i as,

$$P(S|G_i) = \prod_{j=i}^{n-k+1} P(M_j|G_i)$$

Using the naïve Bayesian methodology, the probability of finding sequence S in a certain genome G_i can be calculated as,

$$P(G_i|S) = \frac{P(S|G_i) * P(G_i)}{P(S)}$$

It assigns the sequence S to be originated from the genome G_i with the maximum $P(G_i|S)$ value. In calculation, the probability of find sequence S is a constant, and the prior probability of a genome G_i is the abundance of genomes if the classification strategy is applied to real microbiome samples. As in most case, the abundance is unknown, we assume a uniform distributed abundance that $P(G_i)$ are equal among genomes. Then, the sequence classification procedure is equivalent to the estimated maximum likelihood, $P(G_i|S) \propto P(S|G_i)$.

SVM algorithm in MetaGUN

Feature integration

In MetaGUN, we denote a training item as $\boldsymbol{x}$, which represents the feature vector of an ORF. Given a training set of n items with known class labels, $\{(\boldsymbol{x}_i, y_i)\}_{i=1}^{n}$, where $y_i \in \{+1, -1\}$ (+1 for protein-coding genes, and -1 for non-coding ORFs) is the label associated with x_i, the maximum-margin hyperplane that separates the two classes of the training data is obtained by solving the quadratic optimization problem,

$$\text{minimize}_{(\omega,b,\xi)} \frac{1}{2}\|\boldsymbol{\omega}\|^2 + C\sum_{i=1}^{n}\xi_i,$$

subject to: $y_i \cdot (k(\boldsymbol{\omega}, \varphi(\boldsymbol{x}_i)) + b) \geq 1 - \xi_i$, $\xi_i \geq 0$, for i=1, …, n. The scalar b is the bias, ξ_i is the slack variables that allows the ith instance to be in the margin or misclassified and C sets the relative importance of maximizing the margin and minimizing the amount of the slacks. The procedure of training an SVM classifier is to learn the hyperplane ($\boldsymbol{\omega}$, b) that optimally separates items of two classes in the feature space. The vector $\boldsymbol{\omega}$ is defined as: $\boldsymbol{\omega} = \sum_{i=1}^{n} \alpha_i y_i \boldsymbol{x}_i$, where α_i are weights

⋆ The correspondence author.

assigned to each training item x_i. In the prediction period, an query feature vector $\boldsymbol{x}$ of a candidate ORF can be discriminated by the decision function:

$$f(\boldsymbol{x}) = \sum_{i=1}^{n} y_i \alpha_i k(\boldsymbol{x}, \boldsymbol{x}_i) + b,$$

where $k(\boldsymbol{x}, \boldsymbol{x}_i)$ is the kernel function which is defined as the Gaussian kernel here $(e^{-\gamma\|\boldsymbol{x}-\boldsymbol{x}_i\|^2})$. It calculates if the query $\boldsymbol{x}$ closer to the protein-coding class or the non-coding class by comparing it with all training items. In our method, the regressive version of SVM classifier is trained and employed to estimate the probability of a query ORF to be a protein-coding gene based on the decision value f($\boldsymbol{x}$). An ORF will be predicted to be protein-coding if the regressive probability is larger than 0.5 in both the universal prediction module and the novel prediction module.

SVM classifier training

We implement LibSVM package to train the SVM classifiers and have tried several types of kernel functions, including linear kernel, polynomial kernel and radial basis function (RBF) kernel which is also called Gaussian kernel. Tests showed that the Gaussian kernel leads to the best performance (see Appendix: Supplementary Table 1). In order to train the optimal SVM classifiers for each phylogenetic domain, a grid search of feature space is implemented on a randomly sampled subset of training items to find the most suitable parameters. The size of subset of training items is also tested and we found the results are sufficient good if the size is larger than 5000, we then set the subset size as 10000. During the grid search procedure, different values of the Gaussian kernel parameter γ and the SVM parameter C in the given interval are used to train different SVM classifiers and test the prediction accuracies on the subset of training items. In each step of the grid search, that is, specific given values of γ and C, the 5-fold cross validation strategy is used. Cross validation is a widely used technique to assess the predict accuracy in machine learning methods to avoid overfitting. 5-fold cross validation means that the sample is equally divided into 5 subsamples followed by 5 round performance assessments. In each round, a subsample will be used for evaluating the performance of the classifier trained on the remaining 4 subsamples. After the grid search, the Gaussian kernel parameter and the SVM parameter that lead to the best prediction performance will be chosen to train the final SVM classifier on the whole set of training items. In the training period, all training items from both the protein-coding class and the non-coding class are implicitly mapped from the input space to the feature space determined by the Gaussian kernel under the best γ and C. Then, an optimal hyperplane is learned by the SVM algorithm that separates all the training protein-coding and non-coding items.

The SVM parameter C is the penalty of misclassification when training. If the value of C is large, the margin of the decision hyperplane boundary is relatively 'narrow', which is more like a 'hard margin' since a high C implies the slack variables have high costs. Otherwise, a small C allows the decision plane to ignore the vectors close to the boundary so that increases the margin which makes it 'softer'. Moreover, in the Gaussian kernel function, the parameter γ plays a role in controlling the fitness and the generalization for the classifier, which presented by the flexibility of decision boundary. Large values of γ lead to more smooth and general decision boundary, which is nearly linear. The decision boundary performances greater curvature that is more local along with the decrease of γ. If γ is extremely small, the decision plane becomes discontinuous. It usually means 'over-fitting'. Therefore, in order to train a high accuracy classifier with reasonable C and γ parameters, a grid search is indispensible.

Supplementary Tables

Table 1. Accuracies achieved by universal module with SVM classifiers trained on various training sizes and different types of kernel functions.

Sample size (M)	Linear kernel	Polynomial kernel	Gaussian kernel
0.4	90.0	94.9	95.6
0.8	90.0	95.3	95.7
1.2	90.0	95.3	95.8
1.6	90.1	95.6	95.9
2.0	90.1	95.7	95.9
